# Age-dependent differences in the prognostic relevance of body composition-related variables in type A aortic dissection patients

**DOI:** 10.1186/s13019-021-01742-1

**Published:** 2021-12-28

**Authors:** Zeng-Rong Luo, Xiao-Dong Chen, Liang-wan Chen

**Affiliations:** 1 Department of Cardiovascular Surgery and Cardiac Disease Center, Union Hospital, Fujian Medical University, Fuzhou, 350001 People’s Republic of China; 2 Key Laboratory of Cardio-Thoracic Surgery (Fujian Medical University), Fujian Province University, Fuzhou, People’s Republic of China

**Keywords:** Body composition parameters, BMI, Type A aortic dissection, Age

## Abstract

**Background:**

The current research is allocated to appraise the association between the parameters of body composition and findings in type A aortic dissection (TAAD) cases in diverse age groups.

**Methods:**

Data from consecutive TAAD patients undergoing implantation of modified triple-branched stent-graft from January 2017 and December 2019 were prospectively collected and analyzed. A regression model of Cox proportional hazard was employed to assess correlations among body composition-related variables (body mass index [BMI], lean body mass [LBM], body surface area [BSA], and LBM index) as well as cumulative mortality.

**Results:**

Overall, 258 patients (53.9 ± 11.1 years old; 72.9% male) were separated into young (n = 110) and elderly (n = 148) age groups based upon whether they were younger or older than 50 years of age. Of these patients, 247 survivors were included in subsequent analyses over an average 26.8 ± 11.6 month follow-up duration. Multivariate analyses in the elderly group instead of young group indicated that increased BMI (*p* = 0.042), BMI ≤ 18.5 kg/m^2^ (*p* = 0.025), and lower LBM index values (*p* = 0.019) were significant predictors of increased total all-cause cumulative mortality. BMI was considerably positively correlated with estimated all-cause cumulative mortality in elderly but not young TAAD cases.

**Conclusion:**

Briefly, these results suggest that BMI and LBM indices are only significant predictors of TAAD patient all-cause mortality in elderly patient cohorts, whereas they do not offer significant prognostic value for younger patients. As such, these age differences must be taken into consideration when conducting stratified risk assessments based upon TAAD patient body composition characteristics.

**Supplementary Information:**

The online version contains supplementary material available at 10.1186/s13019-021-01742-1.

## Introduction

Body composition parameters have long been used in clinical contexts to guide treatment-related decision-making and to predict patient outcomes. Body mass index (BMI), body surface area (BSA), and measurements of bone, lean, and fat mass are commonly analyzed variables in this context. Of these, BMI is the most frequently evaluated metric, but in some cases it fails to accurately recapitulate true body fat mass [1]. BSA is thought to be a more robust and reliable predictor of 1-year mortality among individuals with chronic heart failure relative to BMI, as persons with greater overall body size exhibit longer survival irrespective of whether height is corrected for [1]. Lean body mass (LBM) has been realized to independently estimate the incidence of all-cause mortality among coronary cardiac disease cases [2].

Obesity is generally thought to be independently linked to cardiovascular morbidity and mortality risk [3], and some researchers have found that obesity has a negative impact on operative mortality or morbidity among coronary artery bypass graft recipients [4] or valve surgery patients [5]. Paradoxically, however, there appears to be an inverse relation between mortality and body mass [6] among heart failure patients [7], individuals diagnosed with atrial fibrillation [8], and those being treated via percutaneous coronary intervention [9]. This obesity paradox has also been observed for transcatheter aortic valve implantation (TAVI) patients [10]. In some studies, obese cases suffering operative treatment of type A acute aortic dissection (TAAD) were detected to exhibit higher rates of intraoperative death and an elevated risk of post-operative complications, pulmonary complications, and low cardiac output syndrome relative to non-obese patients [11]. However, there have been few reports to date regarding the prognostic relevance of body composition-related variables in TAAD patients. As such, this analysis was designed to examine the predictive value of body composition-related variables within a cohort of Fujian province TAAD cases suffering surgical treatment via an implantation approach of modified triple-branched stent-graft [12].

## Patients and methods

### Patients

In total, this study cohort included 258 patients treated for TAAD from January 2017 to December 2019. All of these patients were diagnosed with TAAD via computed tomography (CT) scans and intraoperative transesophageal echocardiography, and were treated in the Union Hospital of Fujian Medical University via implantation of modified triple-branched stent-graft.

### Outcome measures

Prospective measurements of demographic and body composition-related clinical variables comprising body height and weight were made for all cases prior to TAAD treatment. Appropriate formulas were used to calculate the index values of BSA, BMI, LBM, and LBM for all patients. BMI was calculated for a given patient through dividing their body weight (kg) by the square of their height (m), and was classified using the most prevalent BMI classification system utilized for Chinese adults as follows [13]: normal weight (18.5 ≤ BMI < 25 kg/m^2^), overweight (25 ≤ BMI < 30 kg/ m^2^), and obese (BMI ≥ 30 kg/m^2^). An 18.5 kg/m^2^ BMI cutoff was utilized, as a BMI < 18.5 kg/m^2^ was previously identified as a criterion for frailty among TAAD patients [14]. BSA was computed using the Mosteller equation [15]. LBM was determined with the James equation: for females, LBM = 1.07 × weight [kg] − 148 × (weight [kg]/height [cm])^2^; for males, LBM = 1.1 × weight [kg] − 128 × (weight [kg]/height [cm])^2^ [16]. LBM index values were determined for a given patient by dividing the LBM by the square of their height (m).

Composite primary endpoints for this analysis included peri-operative mortality, permanent neurological damage (paraplegia or stroke), and renal disorder necessitating hemodialysis upon release from hospitalization [17]. Secondary mid-term outcome endpoints included delayed death and a lack of need for delayed aortic reinterventions. Surviving patients were followed via phone calls, letters, or emails as appropriate. CTA was conducted prior to discharge, 3 months postoperatively, and once per year thereafter.

### Procedure details

The surgical processes were executed under general anesthesia as described previously [12, 18, 19] and detailed in Fig. 2a–g and Additional file 1, Additional file 2, Additional file 3, Additional file 4, Additional file 5 and Additional file 6 of the present research. Briefly, during the regular cardiopulmonary bypass process, the ascending aorta was blocked, and incisions were made therein, with proximal operations including sinus reconstruction, aortic valve repair, and ascending aortic artificial blood vessel replacement being completed as appropriate. When the rectal temperature of the patient fell below 25 °C, antegrade selective cerebral perfusion was conducted via the right axillary artery. Then, to expose the true lumen of the descending aorta, an incision was made in the minor curvature of the aortic arch as well as the openings in the three branches of the aortic arch. After arresting circulation, the fundamental segment and branches of the modified triple-branched stent graft were respectively implanted into the true lumen of the descending aorta and the three corresponding branching arteries in the aortic arch, and in turn were released. The stent was then trimmed and two branch stent grafts were fixed as appropriate, after which the innominate artery was blocked and a perfusion tube was inserted into the left carotid artery via the second branch stent graft, and bilateral antegrade cerebral perfusion was performed. The artificial blood vessel was then continuously sutured with the proximal end of the modified triple-branched stent graft, after which whole-body perfusion was restored, patients were warmed, and cardiopulmonary bypass was terminated. The ethics committee of Fujian Medical University, China approved this study.

### Statistical studies

The Kolmogorov–Smirnov method was used to test the normality of the continuous variables. The measurement data with a normal distribution were expressed as means with standard deviations (SD). Categorical variables were given as numbers (percentage). Outcomes between groups were compared via various tests including Student’s t-tests, Pearson’s chi-squared, or Mann–Whitney U depending on the case. A logistic regression model was applied to measure risk scores for each patient based upon baseline variables including height, BMI, body weight, body surface area, LBM, LBM index, age, sex, hypertension, diabetes mellitus, obstructive sleep apnoea syndrome (OSAS), chronic obstructive pulmonary disease (COPD), left ventricular ejection fraction (LVEF), serum creatinine levels, hyperlipidemia, renal dysfunction, malperfusion syndromes, and severe or moderate aortic valve regurgitation (AR). Independent relationships between body composition-related variables and overall or 1-year all-cause cumulative mortality were examined through multivariate Cox proportional hazard regression assessments, the achievements of which were explained as adjusted hazard ratios (HR) with 95% confidence intervals (CIs). The values of BMI were treated as both continuous and categorical variables in the considered models. A two-tailed *p* < 0.05 was the significance threshold, and SPSS 24.0 was employed for statistical studies.

## Results

**Fig. 1:**
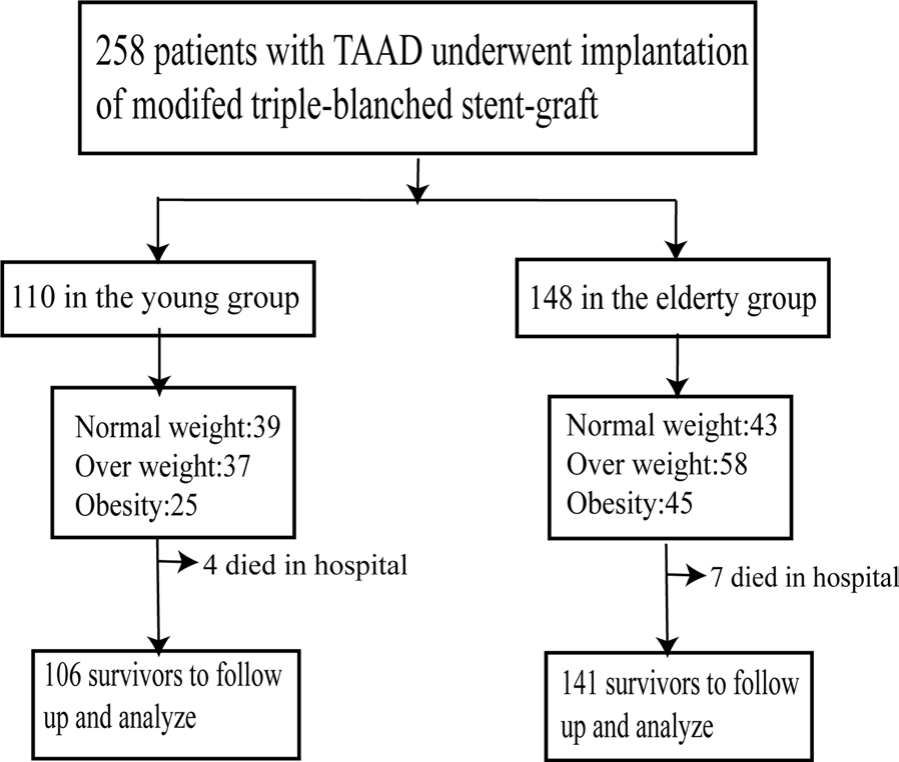
Flow diagram of the screening and enrollment of study patients

Flow diagram of the screening and enrollment of study patients see Fig. 1.

### General patient information

**Fig. 2:**
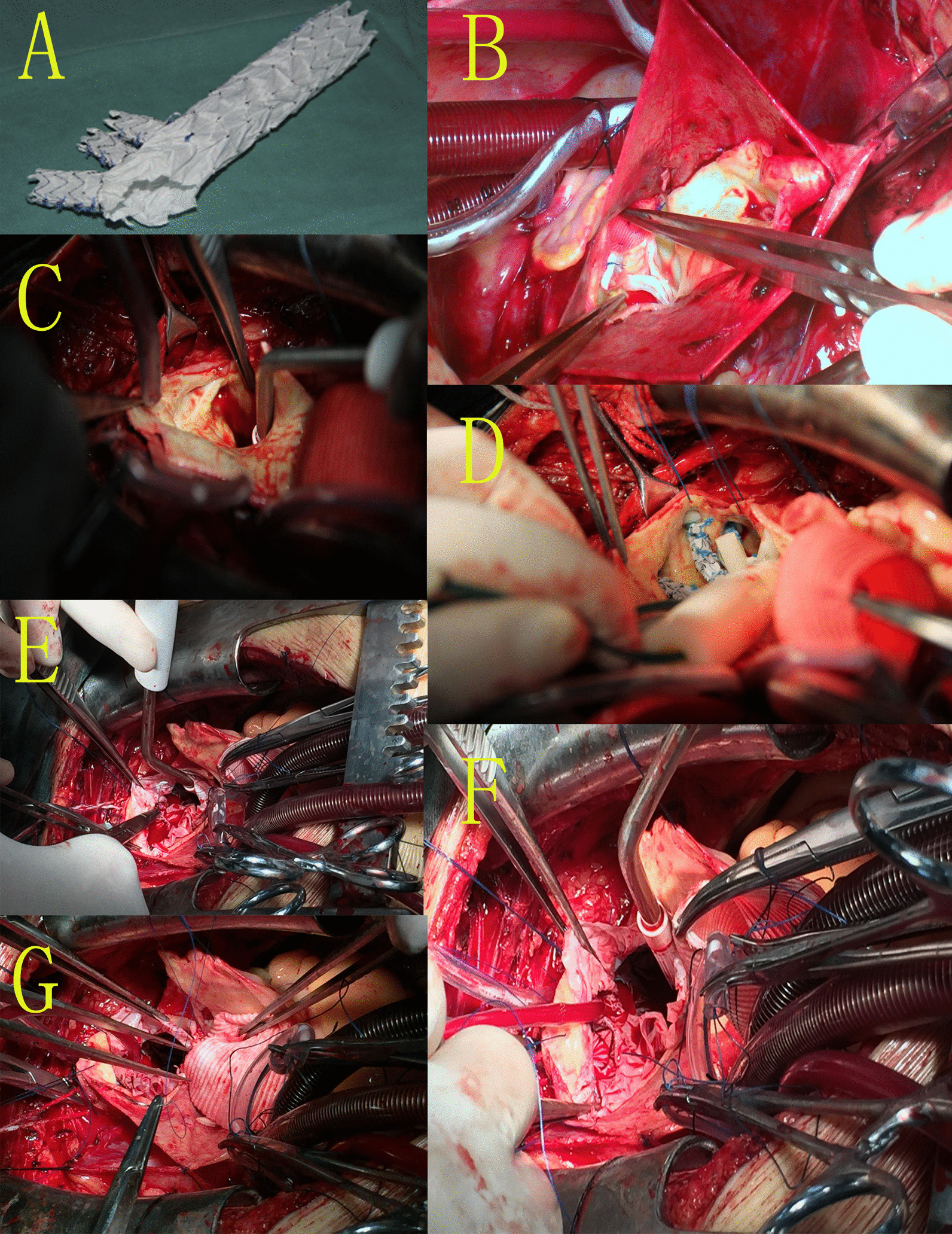
Procedure details of implantation of modified triple-branched stent-graft. **A** Modified triple-branched stent-graft. **B** Aortic root reconstruction and ascending aortic artificial blood vessel replacement. **C** Expose the branching arteries in the aortic arch. **D** Implant and release the modified triple-branched stent-graft. **E** Two branch stent grafts were fixed as appropriate. **F** A perfusion tube was inserted into the left carotid artery via the second branch stent graft, and bilateral antegrade cerebral perfusion was performed. **G** The artificial blood vessel was continuously sutured with the proximal end of the modified triple-branched stent graft

Data were collected and analyzed for 258 TAAD patients with an average age of 53.9 ± 11.1 years, of whom 110 were classified as being young (mean age 43.4 ± 8.4 years, 42.6%) and 148 were classified as being elderly (mean age 59.4 ± 12.6 years, 57.4%). Of the overall patient cohort, 188 (72.9%) were male. The median patient follow-up duration was 26.8 ± 11.6 months. For further details regarding patient demographic characteristics and body composition parameters, see Table 1. There were no considerable discrepancies in gender (*p* = 0.542) or the ratio of obesity (*p* = 0.170) when comparing the young and elderly patient cohorts, whereas the average BMI (*p* = 0.039) and BSA (*p* = 0.031) of the elderly cohort were higher than those of the young cohort, and the LBM (*p* = 0.028) and LBM index (*p* = 0.034) of the elderly cohort were significantly lower than those of the younger cohort. Hypertension rates (*p* = 0.000), diabetes mellitus (*p* = 0.032), and OSAS (*p* = 0.016) were also considerably greater in the elderly cohort relative to the young cohort (Fig. 2).

**Table 1:** Patient characteristics

Variables	All	Young	Elderly	*p*
	(n = 258)	(n = 110)	(n = 148)	
Age (year)	53.9 ± 11.1	43.9 ± 10.4	59.9 ± 13.1	**0.016**
Male	188 (72.9)	78 (70.9)	110 (74.3)	0.542
Body height (cm)	163.9 ± 9.0	167.9 ± 6.5	161.1 ± 8.9	**0.041**
Body weight (kg)	73.5 ± 11.8	77.8 ± 10.8	68.2 ± 13.6	**0.034**
BMI (kg/m^2^)	26.2 ± 4.1	24.8 ± 4.0	26.4 ± 3.9	**0.039**
BMI ≥ 18.5 kg/m^2^	247 (95.7)	101 (91.9)	146 (98.6)	**0.007**
Normal weight	82 (33.2)	39 (38.6)	43 (29.5)	0.275
Overweight	95 (38.5)	37 (36.6)	58 (39.7)	0.360
Obesity	70 (28.3)	25 (24.8)	45 (30.8)	0.170
Body surface area (m^2^)	1.76 ± 0.3	1.80 ± 0.3	1.71 ± 0.2	**0.031**
Lean body mass (kg)	61.4 ± 8.4	63.7 ± 6.3	60.7 ± 4.5	**0.028**
Lean body mass index(kg/m^2^)	19.9 ± 1.7	20.1 ± 1.7	18.3 ± 1.3	**0.034**
Hypertension	216 (83.7)	80 (72.7)	136 (91.9)	**0.000**
Diabetes mellitus	43 (16.7)	12 (10.9)	31 (21.0)	**0.032**
Hyperlipidemia	79 (30.6)	29 (26.4)	50 (33.8)	0.201
OSAS	25 (9.7)	5 (4.6)	20 (13.5)	**0.016**
COPD	26 (10.1)	10(9.1)	16 (10.8)	0.650
Renal dysfunction^a^	60 (23.3)	28(25.5)	32 (21.6)	0.471
Moderate or severe AR	94 (36.4)	41 (37.3)	53 (35.8)	0.809
Malperfusion syndromes	46 (17.8)	21 (19.1)	25 (16.9)	0.648
Cerebral	10 (3.9)	4 (3.6)	6 (4.0)	0.864
Myocardial	10 (3.9)	5 (4.5)	5 (3.4)	0.631
Renal	14 (5.4)	7 (6.4)	7 (4.7)	0.567
Iliofemoral	6 (2.3)	3 (2.7)	3 (2.0)	1.000
Gastrointestinal	6 (2.3)	2 (1.8)	4 (2.7)	0.961
LVEF, %	62.7 ± 6.7	62.9 ± 8.9	61.2 ± 7.7	0.618
Serum creatinine (umol/L)	118.8 ± 98	110.6 ± 88	119.8 ± 108	0.136

Bold values indicate* p* < 0.05

Values are n (%) or mean ± SD

AR, aortic valve regurgitation; LVEF, left ventricular ejection fraction; COPD, chronic obstructive pulmonary disease; OSAS, obstructive sleep apnoea syndrome

Clinical presentation of under weight (BMI < 18.5 kg/m^2^), normal weight (18 ≤ BMI < 25 kg/m^2^), overweight (25 ≤ BMI < 30 kg/m^2^), and obese (30 ≤ BMI < 35 kg/m^2^) patients with aortic dissection Type A

^a^Defined as preoperative creatinine greater than 1.5 mg/dL

### Intraoperative and postoperative data

Different patients underwent a range of concomitant processes including the repair of the aortic valve, the reconstruction of the Valsalva sinus, coronary artery bypass graft surgery, and Bentall procedures, and these procedures were performed at comparable rates in both groups (Table 2).

**Table 2:** Procedural data

Events	All	Young	Elderly	*p*
	(n = 258)	(n = 110)	(n = 148)	
*Concomitant procedures*				
Reconstruction of sinus of Valsava	108 (41.9)	49 (44.5)	59 (39.9)	0.451
Aortic valve repair	31 (12.0)	14 (12.7)	17 (11.5)	0.762
Bentall	41 (15.9)	17 (15.5)	24 (16.2)	0.869
Coronary artery bypass graft	13 (5.0)	6 (5.5)	7 (4.7)	0.792
Mitral valve operation	5 (1.9)	2 (1.8)	3 (2.0)	1.000
Operation time (min)	290.5 ± 87.5	288.5 ± 97.8	290.8 ± 96.8	0.586
Cardiopulmonary bypass (min)	139.8 ± 35.8	138.6 ± 41.6	141.8 ± 35.5	0.558
Cross-clamp time (min)	48.9 ± 18.7	48.8 ± 17.8	49.0 ± 16.6	0.672
SCP and low body arrest (min)	14.1 ± 4.1	13.8 ± 4.8	14.1 ± 6.6	0.614

Values are n (%) or mean ± SD

SCP, selective cerebral perfusion

There existed no discrepancies between groups within the operative duration, duration of cardiopulmonary bypass, cross-clamp duration, duration of selective cerebral perfusion and low body arrest, or mean length of hospitalization between these groups (Table 2).

Postoperative complications include neurological dysfunction, paraplegia or stroke, liver insufficiency, pneumonia, and multiple organ dysfunction syndromes. The 30-day mortality rates in these two groups were 3.6% (4/110) and 4.7% (7/148), with no difference between groups (*p* = 0.906) (Table 3). Elderly patients also exhibited a longer average postoperative ventilation time (*p* = 0.029), and higher rates of pneumonia (*p* = 0.001) and acute kidney injuries (*p* = 0.005) relative to cases in the younger cohort (Table 3).

**Table 3:** Postoperative event rates of clinical outcomes

Events	All	Young	Elderly	*p*
	(n = 258)	(n = 110)	(n = 148)	
Ventilation time (h)	128.2 ± 182.3	120.2 ± 102.2	138.2 ± 100.6	**0.029**
Hospital time (d)	20.6 ± 13.8	20.0 ± 16.8	21.5 ± 15.5	0.529
Complications				
Neurologic dysfunction	9 (3.5)	4 (3.6)	5 (3.4)	1.000
Temporary	6 (2.3)	3 (2.7)	3 (2.0)	1.000
Permanent	3 (1.2)	1 (0.9)	2 (1.4)	1.000
Acute kidney injury^a^	78 (30.2)	23 (20.9)	55 (37.2)	**0.005**
Hepatic insufficiency^b^	76 (29.5)	30 (27.3)	36 (24.3)	0.591
Pneumonia^c^	208 (80.6)	78 (70.9)	130 (87.8)	**0.001**
Multiple organ dysfunction syndrome	8 (3.1)	3 (2.7)	5 (3.4)	1.000
30-day cumulative mortality	11 (4.3)	4 (3.6)	7 (4.7)	0.906
One-year cumulative mortality	16 (6.2)	6(5.5)	10 (6.8)	0.668
Overall cumulative mortality	30 (11.6)	7(6.4)	23 (15.5)	0.023

Bold values indicate* p* < 0.05

^a^Defined as 50% rise in baseline creatinine or new need for dialysis

^b^Defined as bilirubin greater than 5 mg/dL persisting for more than 5 days postoperatively

^c^Defined as positive result in sputum culture requiring anti-infection treatment, or chest roentgenogram diagnosing pneumonia after cardiac surgery

### Follow-up findings

Herein, the 247 surviving patients were followed for 26.8 ± 11.6 months post-operatively (range: 1–36), including 106 and 141 patients in the young and elderly cohorts. Following potential risk factors, the 1-year cumulative mortality rate (5.5% vs 6.8%, *p* = 0.668) did not differ between groups, whereas the all-cause cumulative mortality of the elderly group was significantly increased in comparison to the young group (15.5% vs 6.4%, *p* = 0.023) (Table 3).

**Fig. 3:**
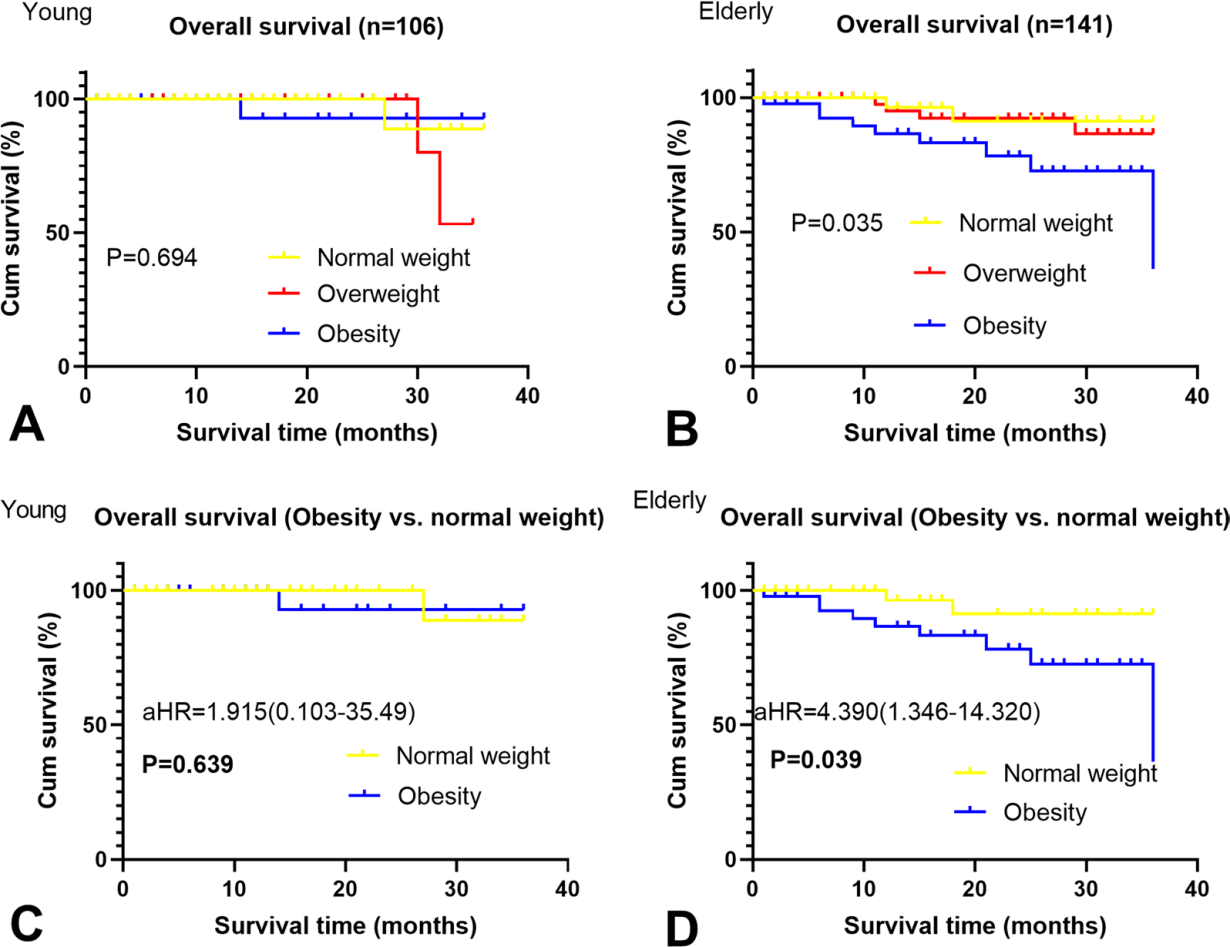
Overall all-cause mortality stratified by normal weight, overweight, and obese for young patients (**A**) and for elderly patients (**B**) and stratified by obesity versus normal weight for young patients (**C**) and for elderly patients (**D**)

**Fig. 4:**
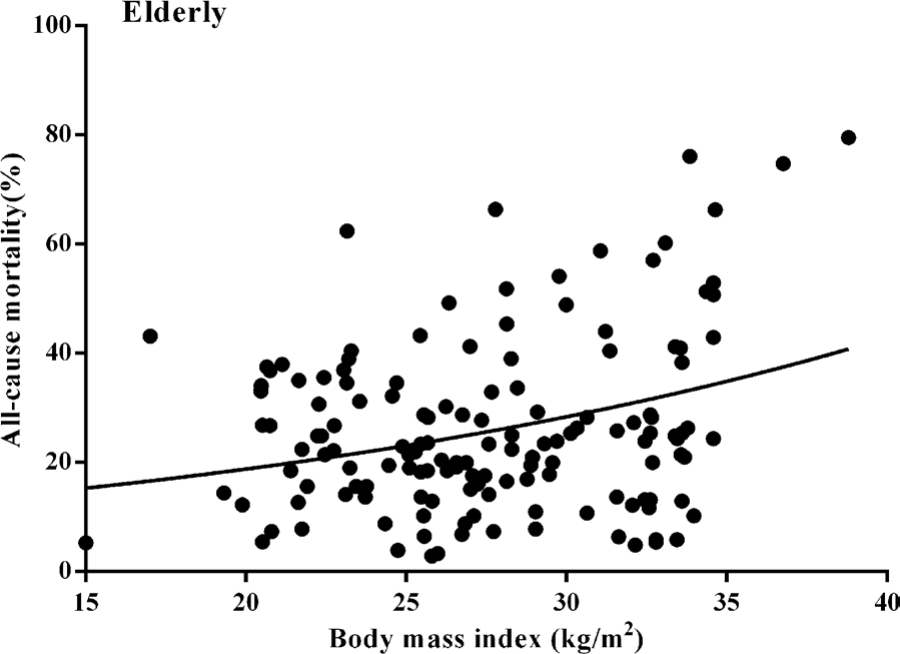
Association between body mass index and estimated all-cause mortality for elderly patients

In the elderly group, BMI (HR: 1.558, 95% CI 0.846–1.988, *p* = 0.042), BMI < 18.5 kg/m^2^ (HR: 0.900, 95% CI 0.116–0.978, *p* = 0.025) and a lower LBM index (HR: 2.668, 95% CI 0.585–2.966, *p* = 0.019) were considerably related to higher total all-cause cumulative mortality, whereas LBM and BSA were not significantly associated with this endpoint (Table 4). The risk for obese patients was significantly increased relative to that of individuals of normal weight (HR: 4.390, 95% CI 1.346–14.320, *p* = 0.039, Fig. 3b, d). No body composition-related variables were considerably correlated with total all-cause cumulative mortality among young individuals (Table 4, Fig. 3a, c), but the significant positive association between predicted all-cause cumulative mortality and BMI was evident only for the elderly patient cohort (Fig. 4).

**Table 4:** Multivariate analysis for overall and one-year all-cause cumulative mortality

Variables	All		Young		Elderly	
	HR (95% CI)	*p*	HR (95% CI)	*p*	HR (95% CI)	*p*
*Overall all-cause cumulative mortality*						
BMI (per 1 kg/m^2^ increase)	1.100 (0.955–1.123)	0.586	1.016 (0.833–1.187)	0.311	1.558 (0.846–1.988)	**0.042**
BSA (per 1 m^2^ increase)	1.148 (0.499–3.699)	0.678	1.306 (0.468–11.886)	0.811	0.551 (0.119–5.558)	0.568
Lean body mass (per 1 kg increase)	0.996 (0.836–1.965)	0.669	1.056 (0.647–2.655)	0.880	1.045 (0.788–1.396)	0.885
Lean body mass index (per 1 kg/m^2^ increase)	1.085 (0.998–1.667)	0.788	1.222 (0.885–1.778)	0.450	2.668 (0.585–2.966)	**0.019**
*Categorical BMI*						
BMI ≥ 18.5 versus < 18.5 kg/m^2^	0.666 (0.466–1.772)	0.660	1.569 (0.336–6.338)	0.668	0.900 (0.116–0.978)	**0.025**
*Categorical BMI*						
Overweight versus. normal weight^a^	0.550 (0.300–1.690)	0.355	1.110 (0.886–3.332)	0.665	0.378 (0.350–0.784)	0.096
Obesity versus normal weight^a^	0.832 (0.611–21.42)	0.565	1.915 (0.103–35.49)	0.639	4.390 (1.346–14.320)	**0.039**
*One-year all-cause cumulative mortality*						
BMI (per 1 kg/m^2^ increase)	0.860 (0.666–1.211)	0.774	1.060 (1.000–2.002)	0.123	1.002 (0.330–1.315)	**0.047**
BSA (per 1 m^2^ increase)	0.411 (0.098–2.228)	0.656	0.888 (0.099–2.115)	0.988	0.858 (0.063–1.215)	0.066
Lean body mass (per 1 kg increase)	0.819 (0.229–1.316)	0.303	1.001 (0.820–1.235)	0.355	1.205 (0.057–1.464)	0.058
Lean body mass index (per 1 kg/m^2^ increase)	1.309 (1.425–2.040)	0.505	1.285 (0.585–1.898)	0.080	1.003 (0.576–1.223)	**0.044**
*Categorical BMI*						
BMI ≥ 18.5 vs. < 18.5 kg/m^2^	0.590 (0.188–1.008)	0.086	1.008 (0.798–3.332)	0.487	0.810 (0.580–1.210)	**0.049**
*Categorical BMI*						
Overweight versus normal weight^a^	0.330 (0.110–1.980)	0.188	0.988 (0.760–1.565)	0.078	0.533 (0.350–0.998)	0.240
Obesity versus normal weight^a^	1.285 (0.318–4.585)	0.169	2.858 (1.085–8.808)	0.081	4.365 (1.268–11.308)	**0.032**

Bold values indicate* p* < 0.05

BMI, body mass index; BSA, body surface area; HR, hazard ratio

^a^Normal weight: BMI < 25 kg/m^2^; overweight: 25 kg/m^2^ ≤ BMI < 30 kg/m^2^; obesity: BMI ≥ 30 kg/m^2^

## Discussion

Prior studies [20, 21] have reported that gender, sex, age, hypertension, left ventricular ejection fraction (LVEF), diabetes mellitus, malperfusion syndrome (MPS), aortic regurgitation, Marfan syndrome, a creatine level > 1.5 mg/dL, and operative duration were all potential risk factors used to assess an early composite endpoint. In our prior study, a multivariate logistic regression analysis indicated that risk factors for an primary composite endpoint for patients undergoing implantation of modified triple-branched stent-graft included MPS (odds ratio 5.17; 95% CI 1.46–18.35; *p* = 0.011) and the levels of creatine > 1.5 mg/dL (odds ratio 5.44; 95% CI 2.27–13.06; *p* < 0.001) [18].

This study is the first to our knowledge to have assessed the prognostic utility of BSA, LBM, and the values of the LBM index as tools for assessing outcomes in TAAD patients undergoing treatment via a modified implantation approach of triple-branched stent-graft. The performed multivariate analysis indicated that BMI, BMI < 18.5 kg/m^2^, and a lower LBM index were all significantly related to increased overall and 1-year all-cause cumulative mortality amongst elderly cases. On the contrary, no body composition-related were linked to all-cause cumulative mortality among younger TAAD patients.

These results may be attributable to the fact that the elderly cases participated in the present investigation exhibited a higher average BMI relative to young patients (26.4 ± 3.9 vs 24.8 ± 4.0), as higher BMI has previously been found to be linked to prolonged ventilation and a higher proportion of hypoxemia following AAD surgical procedures [22]. Acute respiratory disease syndrome (ARDS) is a critical potential complication that could lead to the high rates of pneumonia and prolonged ventilation among TAAD patients, and increased BMI and old age are both known to be preoperative risk factors for ARDS [23]. ARDS incidence has been linked to an imbalance between pro- and anti-inflammatory cytokines and between antioxidant activity and oxidative stress, with most obese cases exhibiting chronic excessive oxidative stress and inflammation [24, 25]. The increased production of reactive oxygen in obese individuals can directly damage cellular membranes, promote the adhesion of monocytic cells, and drive the release of chemotactic factors and vasoactive compounds [22]. Pulmonary function is also commonly impaired in obese individuals, who often exhibit an increase in residual lung volume, reduced ventilatory drive, ventilation-perfusion abnormalities, reduced lung compliance, increases in chest wall impedance, and bronchospasms [26]. Consistent with these prior reports, we observed a longer average duration of ventilation (*p* = 0.029) and higher rates of pneumonia (*p* = 0.001) among elderly patients relative to young patients in this research.

Meanwhile, we found a superior proportion of OSAS (*p* = 0.016), hypertension (*p* = 0.000), and diabetes mellitus (*p* = 0.032) among elderly patients. Nicholl et al. [27] demonstrated a substantial diminution in the glomerular filtration rate (GFR) of the OSAS cases, potentially resulting in renal dysfunction. Indeed, obese individuals have been suggested to be at a higher risk of acute kidney injuries (AKI) owing to the greater rates of comorbidities and potential renal structural alterations, even when their serum biochemistry results are normal [28]. BMI has been found to independently predict AKI following emergency surgical aortic total arch substitute using a frozen elephant trunk implant [29]. A prior multiple logistic regression analysis conducted using data from our center further determined that renal dysfunction was a risk factor for the primary composite endpoint in AAD patients following modified implantation of triple-branched stent-graft [18]. Herein, obese elderly individuals were realized to exhibit an elevated risk of all-cause mortality (HR: 4.390, 95% CI 1.346–14.320, *p* = 0.039) and postoperative AKI (*p* = 0.005), in line with the results of the above reports. Further, hypertension is a dominant risk factor for residual dissection rupture, which may explain the incidence of all-cause mortality in many cases. Diabetes cases are also less probable to appear with rupture of aortic aneurysm or to die as a consequence of aneurysm rupture, indicating that diabetes mellitus may be protective in this context, potentially because of biological modifications in the aortic wall or to indirect benefits derived from secondary prevention or more rigorous monitoring of blood pressure [30]. However, we did not observe such protective effects in the current study, as the rates of diabetes were higher in the elderly cohort relative to the younger cohort, as was the trend in all-cause mortality following TAAD surgery.

We additionally observed a relationship between BMI < 18.5 kg/m^2^ and an increase in overall all-cause mortality rates among elderly patients, in line with prior evidence demonstrating that a BMI < 18.5 kg/m^2^ is an indicator of frailty [31]. To classify frailty, a score was created that consisted of seven components previously identified in published data as objective indicators of frailty. The components were as follows: (1) age more than 70 years; (2) body mass index less than 18.5 kg/m^2^; (3) serum creatinine greater than 1.2 mg/dL; (4) anemia less than 12.0 g/dL for women and less than 13.0 g/dL for men; (5) hypoalbuminemia less than 3.5 g/dL; (6) history of stroke; and (7) psoas muscle area index. One point was given for each criterion met to determine a frailty score of between 0 and 7. Frailty was defined as a score of 3 or greater. Elderly individuals are more physically prone to frailty [32], which has in turn been linked to physiological disorders such as heart rate variability, systemic inflammation, impaired immune functionality, and hormonal changes [33, 34], all of which can contribute to an increased risk of death. In our research, although the mean age in the elderly cohort was far below 70 years old (59.4 ± 12.6 years), the greater number of frail patients among them could explain the higher mortality rate than the young cohort.

Regarding BSA and LBM, in view of the evidence that BSA may be a better measure for investigating physical habits. Muscle is denser than fat, and BMI cannot distinguish between muscle mass and fat mass, which makes it an unreliable indicator of obesity, especially in patients with higher muscle mass. In contrast, BSA approximates obesity by measuring area, so it can better distinguish between muscle and fat [1]. However, we detected no correlation between BSA and all-cause cumulative mortality in young or elderly TAAD patients, which might due to our small sample size and short follow-up period. A prior study of a TAVI patient cohort conducted in Taiwan identified lower LBM as independently predicting increased all-cause mortality among males following TAVI [35]. We observed a similar trend wherein a lower LBM index was closely tied with the enhanced incidence of all-cause mortality (HR = 2.668, 95% CI = 0.585–2.966, *p* = 0.019) among elderly patients following surgery performed via the modified implantation technique of triple-branched stent-graft.

These results of this analysis highlight an age-dependent difference in the correlation between body composition-related variables and the prognosis of TAAD patients treated via a modified implantation approach of triple-branched stent-graft. Overall, our analysis revealed that the 30-day and 1-year all-cause cumulative mortality in the young and elderly TAAD patient cohorts were similar to one another following propensity score adjustment. Differences between these groups may thus be attributable to baseline differences in the demographic characteristics or comorbid conditions in these two populations. Through multivariate analysis, we determined that increased BMI and lower LBM index values were significantly related to an enhancement in overall all-cause cumulative mortality among elderly but not younger TAAD patients, suggesting that these two body composition parameters may be sensitive and reliable indicators capable of stratifying risk in these patient populations.

## Limitations

There exist multiple restrictions to the current survey. First, this was an observational study and it is thus inherently susceptible to potential demographic bias. Additionally, our sample size was too low to permit further subgroup analyses based upon additional age- or BMI-based stratification. During the follow-up baseline parameters and unintended changes in body weight were found to be associated with study outcomes, suggesting that altered physical habits have the impact to influence TAAD patient long-term outcomes, although further research on this topic is warranted. Additionally, owing to a lack of consensus regarding the LBM and LBM index cutoffs, we were unable to classify or categorize these parameters. What is more, various potential confounders such as smoking status, prior cardiac surgery, family history, connective tissue disorder, etc. might have confused the results. Ultimately but most importantly, this was a single-center investigation of a Southeast Chinese population, and additional multi-center studies of populations from multiple countries will be essential to validate these findings.

## Conclusions

In conclusion, our results indicated that BMI and LBM index values serve as significant predictors of TAAD patient outcomes only among elderly patients and not among younger individuals. As such, age differences must be taken into account when conducting stratified risk analyses based upon body composition parameters in TAAD patients.

## Supplementary Information

**Additional file 1**. Ascending aorta and total arch replacement combined with implantation of modified triple-branched stent-graft. Exposure of branch arteries and establishment of cardiopulmonary bypass.

**Additional file 2**. Ascending aorta and total arch replacement combined with implantation of modified triple-branched stent-graft. Aortic root reconstruction and ascending aortic artificial blood vessel replacement.

**Additional file 3**. Ascending aorta and total arch replacement combined with implantation of modified triple-branched stent-graft. Expose the branching arteries, then implant and release the modified triple-branched stent-graft.

**Additional file 4**. Ascending aorta and total arch replacement combined with implantation of modified triple-branched stent-graft. Two branch stent grafts were fixed as appropriate.

**Additional file 5**. Ascending aorta and total arch replacement combined with implantation of modified triple-branched stent-graft. A perfusion tube was inserted into the left carotid artery via the second branch stent graft, and bilateral antegrade cerebral perfusion was performed.

**Additional file 6**. Ascending aorta and total arch replacement combined with implantation of modified triple-branched stent-graft. The artificial blood vessel was continuously sutured with the proximal end of the modified triple-branched stent graft.

## Data Availability

Data sharing not applicable to this article as no data sets were generated or analyzed during the current study.

## Abbreviations

AR: Aortic valve regurgitation
LVEF: Left ventricular ejection fraction
COPD: Chronic obstructive pulmonary disease
OSAS: Obstructive sleep apnoea syndrome
SCP: Selective cerebral perfusion
BMI: Body mass index
BSA: Body surface area
HR: Hazard ratio
